# Student and Faculty Perspectives on the Usefulness and Usability of a Digital Health Educational Tool to Teach Standardized Assessment of Persons After Stroke: Mixed Methods Study

**DOI:** 10.2196/44361

**Published:** 2023-08-10

**Authors:** Judith E Deutsch, John L Palmieri, Holly Gorin, Augustus Wendell, Donghee Yvette Wohn, Harish Damodaran

**Affiliations:** 1 Rivers Lab Department of Rehabilitation & Movement Sciences School of Health Professions Rutgers Newark, NJ United States; 2 School of Graduate Studies Rutgers University Newark, NJ United States; 3 New Jersey Medical School Rutgers University Newark, NJ United States; 4 Art, Art History & Visual Studies Trinity College of Art & Sciences Duke University Durham, NC United States; 5 Informatics New Jersey Institute of Technology Newark, NJ United States

**Keywords:** physical therapy, education, teaching tool, simulation-based learning, computer-aided instruction, standardized assessment, clinical reasoning, sensors

## Abstract

**Background:**

The VSTEP Examination Suite is a collection of evidence-based standardized assessments for persons after stroke. It was developed by an interdisciplinary team in collaboration with clinician users. It consists of 5 standardized assessments: 2 performance-based tests using the Kinect camera (Microsoft Corp) to collect kinematics (5-Time Sit-to-Stand and 4-Square Test); 2 additional performance-based tests (10-Meter Walk Test and 6-Minute Walk Test); and 1 patient-reported outcome measure, the Activities-Specific Balance Confidence Scale.

**Objective:**

This study aimed to describe the development of the VSTEP Examination Suite and its evaluation as an educational tool by physical therapy students and faculty to determine its usefulness and usability.

**Methods:**

A total of 6 students from a Doctor of Physical Therapy program in the United States and 6 faculty members who teach standardized assessments in different physical therapy programs from the United States and Israel were recruited by convenience sampling to participate in the study. They interacted with the system using a talk-aloud procedure either in pairs or individually. The transcripts of the sessions were coded deductively (by 3 investigators) with a priori categories of usability and usefulness, and comments were labeled as negative or positive. The frequencies of the deductive themes of usefulness and usability were tested for differences between faculty and students using a Wilcoxon rank sum test. A second round of inductive coding was performed by 3 investigators guided by theories of technology adoption, clinical reasoning, and education.

**Results:**

The faculty members’ and students’ positive useful comments ranged from 83% (10/12) to 100%. There were no significant differences in usefulness comments between students and faculty. Regarding usability, faculty and students had the lowest frequency of positive comments for the 10-Meter Walk Test (5/10, 50%). Students also reported a high frequency of negative comments on the 4-Square Test (9/21, 43%). Students had a statistically significantly higher number of negative usability comments compared with faculty (*W*=5.7; *P*=.02), specifically for the 5-Time Sit-to-Stand (*W*=5.3; *P*=.02). Themes emerged related to variable knowledge about the standardized tests, value as a teaching and learning tool, technology being consistent with clinical reasoning in addition to ensuring reliability, expert-to-novice clinical reasoning (students), and usability.

**Conclusions:**

The VSTEP Examination Suite was found to be useful by both faculty and students. Reasons for perceived usefulness had some overlap, but there were also differences based on role and experience. Usability testing revealed opportunities for technology refinement. The development of the technology by interdisciplinary teams and testing with multiple types of users may increase adoption.

## Introduction

### Background

Outcome measurement and interpretation using standardized tests are important clinical skills in rehabilitation. The validity and reliability of the assessments are 2 requirements to ensure the appropriate selection and administration of the instruments. Barriers to implementing them in clinical practice include decreased knowledge about the assessment, the lack of time to administer it, difficulty interpreting both the results and psychometric properties, and selecting the correct test for the patient [1-3].

Digital technology in various forms has been incorporated into physical therapy education. Computer-aided instruction was one of the early technologies, using textual, visual, sound, and motion materials to increase the efficacy and efficiency of teaching as well as enhance learning [4]. Computer-aided instruction was initially used as a tool to develop and reinforce knowledge on topic areas such as biomechanics [5], anatomy [6], and orthopedic special tests [7]. Early research findings suggest that computer-aided instruction is comparable with traditional teaching methods [5-11]. In contrast, a recent meta-analysis [12] reported that students who used computer-aided instruction in anatomy education across undergraduate, medical, and other allied health programs outperformed those with classic education in terms of short-term knowledge. Importantly, in a systematic review of physical therapy students, researchers reported that students preferred computer-aided instruction as a supplement to traditional learning [13].

More recently, physical therapy educators have used simulation-based learning experiences to go beyond knowledge acquisition to develop clinical reasoning and critical thinking skills. Simulation-based learning is a technique that is often linked with technology to create guided experiences that may represent the real-world experience [14]. Technologies that may be used to deliver simulation-based learning experiences include mannequins and virtual reality. The literature suggests that simulation-based learning experiences have similar success to computer-aided instruction when implemented in physical therapy curricula. In a systematic review [15], researchers reported that simulation-based learning experiences improved the clinical decision-making, clinical reasoning, and critical thinking skills of physical therapy students when compared with traditional teaching. The proposed benefits of simulation-based learning experiences over computer-aided instruction are that they overcome the lack of patient availability, ensure patient safety, facilitate the role of deliberate practice [16], and solidify learning goals while also assisting students in translating and integrating knowledge into practice [15-17].

To address some of the barriers to implementing standardized assessments and incorporate advances in education technology, the VSTEP Examination Suite—a digital technology—was developed as an education and clinical tool. As learning to administer and interpret standardized assessments requires both basic knowledge and interpretation skill [18], the tool needed to align with clinical reasoning. Therefore, technology developers had to consider the perspectives of the end users—in this case, physical therapist clinicians, faculty, and students.

When developing and implementing a new technology, it is important to assess users’ attitudes toward and acceptance of the technology. Different theoretical frameworks have been used to measure acceptance of technology, and they focus on themes such as usefulness (what are the benefits of using it) and usability (how easy it is to use). The technology acceptance model (TAM) is based on the theory of reasoned action; it measures perceived usefulness, or how technology can improve job performance [19] and usability, and perceived ease of use, or the lack of physical or mental effort required to use the technology. The main focus of the TAM is to predict attitudes toward acceptance of a new technology [19,20]. The Unified Theory of Acceptance and Use of Technology (UTAUT) was created to consider other constructs related to technology acceptance and intention to use technology. In addition to expanding the TAM to include social norms and facilitating conditions, the UTAUT provides a nuanced perspective on usability and usefulness [21]. The UTAUT describes performance expectancy (usefulness) as how useful the technology is to the person in achieving their goals or job and effort expectancy (usability) as how much work one would expect when using the technology. These refined definitions of usability and usefulness are helpful in interpreting attitudes toward technology [22].

### Objectives

The purpose of this study was to develop a set of simulation-based learning experiences for standardized assessments (the VSTEP Examination Suite) and evaluate their usefulness and usability as a teaching and learning tool from the perspective of both physical therapy students and faculty. The evaluation was performed using a mixed methods design emphasizing qualitative data. We anticipated that both groups would find the VSTEP Examination Suite useful but not necessarily in the same way and that usability issues would be identified to guide further refinement of the technology.

## Methods

### VSTEP Digital Health Platform and Examination Suite User-Centered Design

VSTEP is a digital health platform consisting of rehabilitation games and an Examination Suite. The system uses the Microsoft Kinect One camera (Microsoft Corp) to capture kinematics and provide visual displays to facilitate administration and interpretation. The software was written in Unity 3D (Unity Technologies) and uses JSON files to define the structure and flow of the software as well as the format of the results tables. There is a patient intake graphical user interface that collects information about the individual’s demographics, health condition, and use of assistive devices or orthotics. The platform was developed originally for use in clinical practice [23] and then considered as a tool for teaching. The platform was developed based on a user-centered design with input from clinicians [24].

The VSTEP Examination Suite is an evidence-based battery of standardized assessments of balance, mobility, coordination, and balance confidence designed for persons after stroke. Standardized assessments are organized into categories (eg, balance, mobility, and composite, addressing multiple body function structure elements). Each test has a setup, test administration diagram, instructions, results, and comment screens. The assessments were selected in several phases. First, the Stroke Evaluation Database to Guide Effectiveness II task force recommendations for persons with stroke [25] were reviewed, and tests (eg, 5-Time Sit-to-Stand [5XSTS]) that would be feasible to acquire using the Kinect camera were identified. Second, consultation with clinicians (n=7) in a large rehabilitation center and a university-based practice (n=4) identified tests commonly used in their clinics for the assessment of persons after stroke. Third, alignment with the clinical practice guidelines for outcome measures in persons with neurologic health conditions was considered [26]. A total of 5 standardized assessments were selected: 2 performance-based tests using the Kinect camera to collect kinematics (5XSTS and 4-Square Test [4SQT]); 2 performance-based tests (10-Meter Walk Test [10MWT] and 6-Minute Walk Test [6MWT]); and 1 patient-reported outcome measure, the Activities-Specific Balance Confidence Scale (ABC). The instructions for the tests followed the clinical practice guidelines on outcome measures [26].

**Figure 1:**
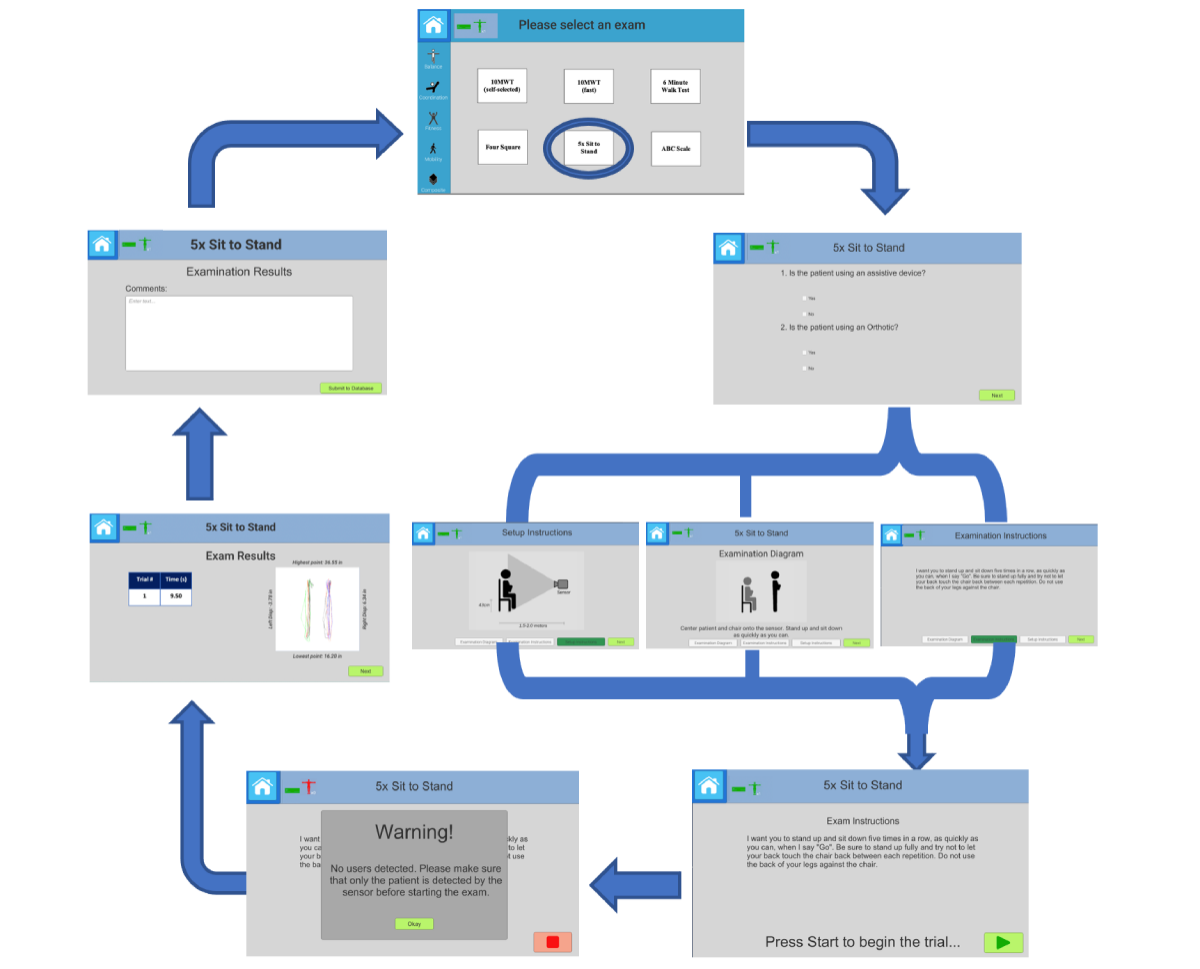
A flow diagram of the VSTEP Examination Suite shows the progression of the 5-Time Sit-to-Stand (5XSTS) test. The progression flows from test selection (top), to setup and test instructions (right side), and to test results and termination (left side). Three images related to test setup, diagrams, and instructions are linked on the right side as the user can freely navigate among these 3 screens as needed. After answering the questions about prosthetic devices and orthotics, users transition directly to the “Examination Diagram” screen. Users can then skip any of the screens and move directly to the “Start Exam” screen (bottom right) by selecting “Next” on either of the 3 screens. For example, the user can skip the “Setup Instructions” screen if the test is already setup or skip the “Examination Diagram” if they are familiar with the examination. The exam results screen illustrated the kinematics (anterior superior iliac spine movement tracked) during sit-to-stand as well as the time. 10MWT: 10-Meter Walk Test.

Wireframes (an image that displays the functional elements of a page) were developed for each of the tests with a proposed sequence for navigation. Focus groups with clinicians who had experience working with persons after stroke across multiple settings (outpatient university-based clinic, free-standing inpatient facility, stroke unit, and home care) solicited feedback on image clarity and consistency of the workflow with their clinical reasoning. These focus groups were 1.5 hours long. Wireframes were iterated and revised with the 2 original focus group participants, who offered additional feedback with an emphasis on ease of use and alignment with clinical reasoning. Changes to the software included revising unclear images, changing navigation buttons that were confusing, and correcting technical glitches (eg, the results of the test did not populate). Both focus groups were facilitated by an investigator experienced with the technology and conducting usability studies (JED) and a second observer also familiar with the technology and user studies (HD). Both the facilitator and the observer documented the comments made by the participants in writing and compared their notes to make the revisions to the wireframes. The version of the VSTEP used in this study was the product of this early phase of development. An example of the sequence of graphical user interfaces for administering and interpreting the 5XSTS is presented in Figure 1.

### Procedure

#### Recruitment

Faculty participants were recruited (via email) from universities in the United States and Israel, and students were recruited (via announcement in class) from Rutgers University. Students were purposively recruited in their last academic semester to ensure that they had received all the relevant instruction in standardized assessments and completed 1 clinical rotation. A total of 7 faculty members who taught standardized assessments and 7 students were recruited, and 86% (6/7) agreed to participate in each group. There were no dropouts from this study.

#### Testing Protocol

Faculty and students were tested separately. Data were collected in a university setting. A facilitator and 2 additional investigators were present for all tests. The facilitator (JED) oriented the participants to the system features (eg, how to know if the camera was recording and basic navigation) and the organization of the VSTEP tests. Participants, either individually or in pairs, used the VSTEP Examination Suite and executed the ABC, 5XSTS, 4SQT, 10MWT, and 6MWT. They alternated assuming the role of the “patient” performing the test and the “clinician” administering the test. Audio data were collected using a talk-aloud procedure guided by an outline of preselected questions (Multimedia Appendix 1). Questions were pilot-tested with students not included in the study. At the end of the 1-and-a-half–hour sessions, the participants were debriefed.

#### First Round of Qualitative Analysis

Audio recordings were transcribed and entered into NVivo Pro (version 12; QSR International). Content analysis was performed using deductive reasoning with 3 a priori categories—usefulness, usability, and suggestions. In total, 3 investigators performed the coding concurrently using a common set of definitions and clarifying the coding rules.

Usability was operationally defined as comments made by the participants regarding the ease of use of the technology (eg, graphical user interface organization and esthetics, clarity, ease of use, difficulty of use, technical use, technical glitches, and navigation). Usefulness was operationally defined as comments made by the participants about the perceived value of the technology (eg, examples of the program’s application or relevance to education or practice). Observed behaviors consistent with best practices for test administration (eg, relating the test to a patient case) were also considered under the category of usefulness.

Comments were also assigned a positive or negative value as follows: (1) a positive value was assigned if participants stated that they liked an aspect of the software or if they responded in a manner that reflected comprehension without the need for explanation by the testing administrator, and (2) a negative value was assigned if participants stated that they were confused or it would be confusing or not useful for others and if they asked questions for clarification or behaved in a way that required further explanation.

#### Quantitative Analysis

Frequency counts were generated for both the positive and negative usefulness and usability codes. Data were assessed for normality using a Shapiro-Wilk test. As assumptions of normalcy were not met, analyses of differences in the frequency of negative usability and usefulness codes between faculty and students were analyzed using 2-sample Wilcoxon rank sum tests (α=.05). Data were inspected visually, and 2 post hoc comparisons were performed and corrected for the number of comparisons (α=.025).

#### Second Round of Qualitative Analysis

A second round of qualitative analysis was performed concurrently by 3 raters by generating summary statements based on the a priori categories of usefulness and usability. Emergent themes were generated independently by 3 raters using inductive reasoning of the summary statements [27,28]. Final themes were derived from discussions in which the 3 raters reached an agreement. The inductive process was guided by the taxonomy by Bloom [18], clinical reasoning frameworks [29-32], the TAM [19], and the UTAUT [21]. The resultant themes reflect both usefulness and usability in the context of the theoretical frameworks. Usefulness was further divided into the categories of teaching and learning, role of technology, and clinical reasoning.

Themes and representative quotes were sent to the faculty. They were asked to either agree or disagree with the themes and the specific quotes that were attributed to them. There was 100% agreement among faculty members. The students had graduated and were not contacted.

### Ethics Approval and Informed Consent

This study was approved by the institutional review board of the New Jersey Institute of Technology (approval 2007000636). All participants were consented.

## Results

### Quantitative

Faculty participants (n=6; 5/6, 83% female; aged 40-63 years) taught outcome assessment and worked with students in university-based clinical classes and a pro bono clinic in different physical therapy programs in the United States and Israel. Student participants (n=6; 3/6, 50% female; aged 23-42 years) were in their last semester of academic preparation of a Doctor of Physical Therapy program in the United States.

The faculty members’ positive usefulness comments ranged from 83% (10/12) to 100%. The students’ positive usefulness comments ranged from 92% (12/13) to 100%. There were no significant differences between faculty and students regarding the frequency of positive or negative usefulness comments (Multimedia Appendix 2).

The faculty and student positive usability comments ranged from 50% (5/10) to 100%. Usability scores varied by test; for both groups, the number of negative usability comments was greatest for the 10MWT, followed by the 4SQT and the 5XSTS. Students had a statistically significantly higher number of negative usability comments compared with faculty (*W*=5.7; *P*=.02). Students had significantly more negative usability comments than faculty on the 5XSTS (*W*=5.3; *P*=.02). Negative usability comments for the 4SQT approached significance (*W*=4.1; *P*=.04; Table 1), but this result was not statistically significant.

**Table 1:** Positive and negative usability comments by students and faculty.

	Students comments (n=69), n/N			Faculty comments (n=52), n/N (%)	
	Positive usability comments (n=47; 68%)	Negative usability comments (n=22; 32%)^a^	Positive usability comments (n=46; 88%)		Negative usability comments (n=6, 12%)
10MWT^b^	5/10 (50)	5/10 (50)	2/4 (50)		2/4 (50)
4SQT^c^	12/21 (57)	9/21 (43)	17/20 (85)		3/20 (15)
5XSTS^d^	14/21 (67)	7/14 (33)^a^	13/14 (93)		1/14 (7)
6MWT^e^	6/6 (100)	0/6 (0)	2/2 (100)		0/2 (0)
ABC^f^	10/11 (91)	1/11 (9)	12/12 (100)		0/12 (0)

^a^*P*=.02; higher frequency of total and 5XSTS negative usability among students.

^b^10MWT: 10-Meter Walk Test.

^c^4SQT: 4-Square Test.

^d^5XSTS: 5-Time Sit-to-Stand.

^e^6MWT: 6-Minute Walk Test.

^f^ABC: Activities-Specific Balance Confidence Scale.

### Qualitative

A total of 5 main themes emerged from the inductive analysis: previous knowledge, value as a teaching and learning tool, role of technology, clinical reasoning, and usability. Faculty and students had similar themes but different observations within the themes. For example, the perceived value as a teaching tool for the faculty was based on aspects related to the administration of the teaching process, whereas the students commented on the visual representations aiding with recall. Faculty and student themes from the inductive analysis are presented in Table 2. The quotes are associated with the relevant theories. Supporting quotes from the faculty are presented in Textbox 1, and supporting quotes from the students are presented in Textbox 2.

**Table 2:** Themes and representative sample statements for faculty and students.

Theme	Faculty	Students	Theory
Knowledge of standardized tests	Variation in recall influenced administration	Inconsistent recall of tests and where they were taught and practiced in the program	—^a^
Perceived value as a teaching and learning tool	Instructions for tests Bundling of materials and documentation Interpreting results (clinical meaning and movement pattern) Patient explanations Role of comments in interpreting test modification or failure	Visual representation of tests (recall device) Experiential learning	Taxonomy by Bloom [18]
Technology	Software promoted clinical reasoning: Graphical presentation Normative values Comment feature Assistive device Sequential results Tracking patient over time Camera changes the way you perform the test Ascribed features to the system that it does not currently have but that are planned, such as the following: Counting laps Automatic starts Promotes reliability of testing: Standardized instructions provided before every exam Setup diagrams with distances labeled Cue for retesting patients	Wireframes and design were consistent with clinical reasoning: Instructions Images of tests Graphical representation of results Normative data	TAM^b^ (usefulness) UTAUT^c^ (perceived usefulness)
Clinical reasoning	See “Technology”	Exhibited novice clinical reasoning: Details of test Details of setup Exhibited expert clinical reasoning: Patient-centered Movement observation and interpretation	Taxonomy by Bloom [18] Novice to expert
Usability	Recognizing usability concerns but moving past them quickly Intuitive organization and ease of administration	More distracted by negative usability Mixed valence about images of tests and clarity of results (graphs of movements)	TAM (perceived ease of use) UTAUT (effort expectancy)

^a^No specific theory.

^b^TAM: technology acceptance model.

^c^UTAUT: Unified Theory of Acceptance and Use of Technology.

**Themes and representative sample statements for faculty.**

**Knowledge of standardized tests**

- Variation in recall influenced administration
  “...The only other thing is I forget actually is what it says to do. Does it say it gets completed on the final descent?” [Faculty 6; 5-Time Sit-to-Stand (5XSTS)]
  “...Maybe it’s not relevant so much of the scale in terms of how the skill is scored. Is there an option to choose not applicable?” [Faculty 5; Activities-Specific Balance Confidence Scale (ABC)]

**Perceived value as a teaching and learning tool**

- Instructions for tests
  “Yeah I think in general the instructions are a very important thing to practice because a student doesn’t get why it’s important and they think they can say whatever they want. Sorry it’s my like general impression they are not so serious about instructions.” [Faculty 4; 6-Minute Walk Test (6MWT)]
- Bundling of materials and documentation
  “Yeah and I liked it at the you know you have all the information within one source and it’s here and it’s every time and it’s nice. It’s nicer than this piece of paper.” [Faculty 4; 6MWT]
- Interpreting results: clinical meaning, normative values, movement pattern, and patient explanations
  “And also then to interpret you know looking at the interpretation of the data and one relative to the other if there are differences there’s no differences is usually the 6-minute walk there are some differences between students so this can be something that could be more fun.” [Faculty 4; 6MWT]
  “I don’t know but you know we have on clipboards all the normative data because people want to know they want to know how did I do compared to others and it’s a good education talking point as well so that’s really advantageous here.” [Faculty 6; 10-Meter Walk Test (10MWT)]
  “So I could say ‘well this is what a person without a stroke looks like and this is the normative data for a person with a stroke. But this is how you look.’ You know in a positive way at least to say [to the patient] you know you fall you know you’re not the normative for persons with stroke you’re doing a little bit better but you could still see like what if he had this deviation like let’s look there a little bit further let’s look to rehabilitate that you know? So that it’s always done and how can we remediate it.” [Faculty 2; 4-Square Test (4SQT)]
  “This isn’t going to be a full test but I’m thinking ‘what if do a lab practical test and I have the students do the four-square step test I had them interpret the results to the patient.’” [Faculty 3; general]
  “So the normative would actually tell you how to interpret it somewhat like to the clinician.” [Faculty 2; 5XSTS]
- Role of comments in interpreting test modification or failure
  “So as a teaching tool I guess you’re going to have a section where you could fill out what the reject is for right? So if you reject it like I stepped over the cane or I missed a section okay. You would just have an open comment field? Cool.” [Faculty 3; 4SQT]

**Technology (1)—promoted clinical reasoning**

- Graphical presentation of results (all comments made while reviewing the results screen)
  “Oh my could you think about knowing if I—because as a clinician you’re always trying to get somebody to have equal weight-bearing. Yeah that’s cool. Really neat.” [Faculty 3; 5XSTS]
  “I mean I think having this representation visually is probably more helpful than this it just and I realized that in a stroke or in a population which there is a symmetry that deviation can give you kind of good information on performance but that’s quite incredible to be able to look at the pair and sort of the foot placement in each Square because the direction keeps changing.” [Faculty 6; 4SQT]
  “Yeah yeah yeah that’s awesome. [saving feature] So then when you save it and then when you want to test a patient again you can say—‘look where you were and where you were going.’” [Faculty 3; 4SQT]
- Comment feature
  “I think it’s always helpful to have a comment.” [Faculty 6; 5XSTS]
- Assistive device and comment feature
  “I think it’s important yep [to note assistive device]. So, let’s say that I started my therapy session and I worked on walking with a walker and then as the patient got better I wanted to compare how is their self-selected speed with a walker versus a cane so by asking is a patient using an assistive device you are yes or no and then I may want some type of qualifier but I could just throw that in the comments.” [Faculty 3; 10MWT]
- Tracking patient over time
  “Yeah, I think that would be brilliant actually. Because right now when we do all these tests on paper or even if we did it in the outpatient setting, where I do it, I have to pull up week 1 and then it doesn’t do it for me so that would be great if you could program it. You know you always usually have a printout in the patient’s charts I open up the patient chart and I show them this was your ABC on week one but I have to pull it out.” [Faculty 2; ABC]

**Technology (2)—camera changes the way you perform the test**

- Ascribed features to the system that it does not currently have but that are planned, such as automatically detecting the start and end of the test and tracking laps
  “it’s a really good idea [count laps] It’s way more complicated than you think.” [Faculty 6; 6MWT]
  “Yes so—but do I have to take time or just press the start? Can you use the Kinect in any way to recognize the start and end?” [Faculty 4; 10MWT]
  “The only other thing is I forget actually what it says do they say gets completed on the final descent?...No no no, not if it’s being done by the camera...You do it okay yeah.” [Faculty 6; 5XSTS]

**Technology (3)—promotes reliability of testing**

- Standardized instructions provided before every exam
  “Yeah I think in general the instructions are very important thing to practice because a student doesn’t get why it’s important and they think they can say whatever they want. Sorry it’s my like general impression they are not so serious about instructions.” [Faculty 4; 6MWT]
- Setup diagrams with distances labeled
  “Yeah I think if you want to keep like with the same distance and everything it makes sense to have it kind of all set up together so I think that makes perfect sense even though it’s not being recorded it makes sense. Cool okay. One full lap makes sense [reading directions].” [Faculty 3; 6MWT]
- Cue for retesting patients
  “And actually I think a lot of times with the outcome tools in the clinic we use an outcome tools student use it because our students change shifts and everything so it’s a different clinician next week so student X will see me this week but you’ll see me the next week—they don’t think to retest but maybe the patient is also checking saying I can see like Patient X saying ‘are you going to test me on that thing again because remember you know?’ Now not all patients will remember to get retested but it is a nice check and balance and then it’s also that report in your chart you know? Where it’s here in the patient’s electronic record there’s a little trigger that says ‘boop boop it’s two weeks you’ve gotta retest Patient X and see how he does this week.’” [Faculty 2; ABC]

**Clinical reasoning**

- See Technology (1)

**Usability**

- Recognizing usability concerns but moving past them quickly
  “Yeah I don’t know if you want too many visuals I think just like home maybe we’ll just like a little sticky like this it just says home square that’s fine you know just a little something.” [Faculty 2; 4SQT—negative valence]
- Intuitive organization and ease of administration
  “Actually. I think it's very, very nice also for practice so if you can use the same application here and just let the patient practice and then at the end you have this presentation—representation of what they did and you can show him as a feedback.” [Faculty 4; 5XSTS—positive valence]

**Themes and representative sample statements for students.**

**Knowledge of standardized tests**

- Inconsistent recall of tests and where they were taught and practiced in the program; tests introduced but not always practiced in class
  Tester: “Are you familiar with the ABC?”
  Student 4: “I think so.”
  Student 3: “Yes we learned about it in neuro.”
  Tester: “And did you actually administer it to each other and score it?”
  Student 3: “Um I don’t think so. I know it was recommended like in the CPG, that was one of the one’s recommended in the CPG...or is it by APTA?”
  Tester: “Okay so you have one experience physically practicing the 4SQT. Was it in Examination & Measurement?”
  Student 6: “Yeah.”
  Tester: “And did you practice it since then?”
  Student 6: “No.”
  Student 5: “I thought we did it in neuro maybe.”
  Student 6: “Did we? I don’t remember it.”
  “I don’t think we ever did it, we might’ve?” [Student 4; 4-Square Test (4SQT)]

**Perceived value as a teaching and learning tool**

- Visual representation of tests and test results (recall device)
  “It’s also for the patient too yeah that we can administer this a lot and we’ll start to memorize the questions but if it’s their first time taking it, they don’t remember all the questions [of the ABC] they answered so having them see it too would be good.” [Student 5; Activities-Specific Balance Confidence Scale (ABC)]
  “I feel like when we were first learning gait and to see it—it takes time and practice to watch people’s gait and learn if you have some sort of technology to map that and then you can see it that would be that would be—I think—help the learning process and obviously in clinic it’s going to show how the patient’s moving and what they’re doing and how you can help them.” [Student 5; 6-Minute Walk Test (6MWT)]
- Experiential learning
  “Yeah definitely. I think that it solidifies the test just because there’s so many tests that we have to get up and go walk and come back and it is distinctive. Like oh this is the one where you have to stop at the 2-meter mark and start at the 2-meter mark. think it’s a good test.” [Student 4; 10-Meter Walk Test (10MWT)]
  “Not everybody gets a chance to do everything I think it’s because of their groups are so big sometimes and there’s not enough TAs so some people are just watching. I think doing it is what helps you remember what’s what and also helps you remember what things are for like this is for Fitness right so it’ll help you with that and it has like the category too which I think would help the student remember if I want to test Fitness I can do the 6-minute walk test so I like the whole program it’s organized it’s interesting to look at it’s helpful.” [Student 4; 10MWT; general]
  “I think anything is better than paper sometimes. More active learning everything on paper and then doing it with a partner.” [Student 5; 4SQT]
  “Yeah. I do kind of like that this idea this system can guide you through administering the test as well as having you feel like the patient is actually performing it, and I think that’s a good way to teach it because in lab when you have the teacher explain it and then you break up into groups and everyone has questions I think actually having this as a tool in there might help with that.” [Student 6; 4SQT]
  “Yeah especially the first year you know as a confidence thing too. Its ability to build confidence in what you’re doing and that’s—any opportunity to do that is good.” [Student 5; ABC]

**Technology: wireframes and design were consistent with clinical reasoning**

- Instructions for test
  No specific quotes as students responded “yes” when asked if having the instructions was useful or demonstrated that they understood instructions by correctly performing the test
  “Yeah okay so you’re going to walk at your own comfortable pace and you’re going to stop when you get to this last cone over here okay? And then back here.” [Student 6; 10MWT]
- Images of tests
  “I think what was represented was clear enough because there’s a little more involved with like the walking ones but like the five times sit-to-stand is pretty self-explanatory. ABC is pretty self-explanatory. The Four Square you have like the diagram.” [Student 6; 6MWT]
  “Yeah this is actually really helpful. Because I remember when we were actually administering this without this thing it was very unclear where the markings were supposed to be.” [Student 6; 10MWT]
- Graphical representation of results
  “Sort of like feedback for the patient and so they can sort of see—like they have visual representation of how they’re moving. Like they can’t—if they’re looking up the entire time they might not be able to feel that they’re deviating a certain way but by looking at this they’ll know okay I did go that way.” [Student 6; 4SQT]
  “Like if neglect or in Pusher syndrome they might not recognize that they are you know neglecting one side or deviating and this would show them that.” [Student 5; 4SQT]
  “I like having a visual representation of how they’re moving because they themselves might not understand. They might not—they could feel it’s kind of happening or they could feel off balance or something or they could be having difficulty but to see it mapped out might be more beneficial and then to have them actually see the chart where they could compare it to their age group for their diagnosis.” [Student 5; 5-Time Sit-to-Stand (5XSTS)]
- Normative data
  “Even for them [patients] too. If they’re stubborn and they think that they’re safe and it shows that they’re not safe if you could use that as a point of discussion.” [Student 5; 10MWT]
  “And then having normative data underneath the final screen I think I just really like normative data I like comparing things and seeing where your status is at that time.” [Student 4; general]

**Clinical reasoning**

- Exhibited novice clinical reasoning
  Details of test administration “I wasn’t watching for quality of movement I was just watching to count and make sure he was done with 5.” [Student 1; 5XSTS]
  Details of setup (of the sensor for 5XSTS) Student 6: “Does it matter what the height of the sensor is or no?” Student 5: “Because it’s kind of vague in the picture.” Student 6: “Yeah like some kind of a range of the height would help.”
- Exhibited expert clinical reasoning (see also Technology and Clinical Reasoning)
  Patient-centered “Not really nothing I’m thinking about some of like the stroke patients that I did work with I think that it would have been a nice like something different for them to do something for them to look at and see that they’re getting better like a lot of them would like I feel bad saying this but they would cry to me like about how they how they used to be able to do so much because they were young and I think it would be good for them to see their progress visually rather than just have somebody telling you constantly that you’re getting better.” [Student 4; general] “Even for them [patients] too. If they’re stubborn and they think that they’re safe and it shows that they’re not safe if you could use that as a point of like discussion.” [Student 5; 10MWT] “I’ll probably talk about how like this test will probably explain what the test is for to measure balance and then based on how they did I’d explain that their balance isn’t very good and because of that they have a higher risk of falling down at home so we have to work on balance so that they don’t fall down I feel like.” [Student 4; general] “I do like it for the patient to be able to see kind of how they’re moving [graphical representation of test result].” [Student 6; 5XSTS] “I think just you know keeping it consistent between patients and using something that’s in literature and pulling it into clinical practice was great.” [Student 5; 10MWT]
- Movement observation and interpretation
  “Yeah I would say so especially if somebody...say you’re reassessing and all of a sudden you see this deviation I’d want to pull that and see what was going on were they favoring the right or whatever and helping you figure it out.” [Student 2; 5XSTS]
  “I guess maybe if it was like somebody who was leaning to the right or something it would be good for them to see visually that they’re this way [lateral displacement] versus up and down.” [Student 4; 5XSTS]

**Usability**

- More distracted by negative usability than faculty
  “Can you go back to the okay wait, I think the instructions we learned for this was no arms right so arms across and especially if there’s a chair with armrest they’re not allowed to push should that be incorporated?” [Student 2; 5XSTS—negative valence]
  “I mean I guess even if you just said like oh these points are your hips from the lowest point to the highest point. And I guess if I did have if it wasn’t so symmetrical maybe I would understand it more right away?” [Student 3; 5XSTS—negative valence]
- Mixed positive and negative comments about images of tests and clarity of results (graphs of movements)
  “I feel like if I was a patient I would just look at the time because it’s just very basic and that’s what you see. But, like, I don’t really know what the graph—what I’m exactly looking at I guess.” [Student 3; 4SQT—negative valence]
  “I think that’d be great that they’re [footfalls of the 4SQT] in different colors so you can tell which one is which.” [Student 5; 4SQT—positive valence]
- Positive comment for navigation
  “Its simplicity. The multiple ways you can sort of adjust the things. Clicking on the bar and you have the marks for like each 10 or you can slide it. Questions are pretty much exactly the same.” [Student 6; ABC—positive valence]

## Discussion

### Principal Findings: Usefulness and Usability Themes—Comparison of Student and Faculty Perspectives

Both faculty and students using the VSTEP Examination Suite determined that it was useful. Positive comments on usefulness from both groups across the tests ranged from 83% (10/12) to 100%. Students identified more negative usability issues than faculty, especially for the 4SQT (9/21, 43% negative comments from students compared with 3/20, 15% from faculty). Most of the negative usability comments were about timing and executing the test properly (10MWT), interpreting the movement graph (4SQT), and understanding the images of the test instructions (5XSTS). Themes emerged from the qualitative inductive analysis that facilitated the interpretation of the usefulness category and provided more details on usability. The themes will be elaborated on in interpreting similarities and differences between students and faculty.

Both faculty and students exhibited varied recall of the standardized assessments. Students had different recollections of where they learned and practiced a test. Students with experience in the pro bono clinic, where standardized assessments were routinely used, and those who had some exposure during their clinical experience appeared to have greater confidence with the tools. This is consistent with context offering meaning for learning [33] as well as opportunities to practice. Faculty members were generally familiar with the tests, but they also exhibited some variability in their recollection, specifically in how to score the ABC items when a patient does not customarily perform the activity. One faculty member (Faculty 3) was actively involved in knowledge translation for standardized outcome measures. Her recall was in line with the clinical practice guidelines on outcome measures [26], specifically for the 5XSTS, which can be performed without arms crossed for persons after stroke to accommodate the lack of upper limb control [34]. However, other faculty members executed the test with their arms crossed in front of their chest. The variable test recollection even for faculty who taught the content suggests that tools to teach test administration and interpretation may enhance teaching and learning. This is further supported by the faculty theme of how technology supported the reliability of administration (see Textbox 1 for faculty comments on reliability).

Although both groups found the VSTEP Examination Suite to be valuable as a teaching tool, their perspectives on why it was valuable differed. Faculty members appreciated the single location of all the tests and the storage of information (see comments by Faculty 4 on the 6MWT; Textbox 1) This feature reduced their setup for teaching and enabled them to track student performance. They also appreciated the opportunity to highlight and reenforce important concepts such as the validity and reliability of the tests and concepts of clinically meaningful differences (see comments by Faculty 6 on the 10MWT; Textbox 1).

The students, in turn, focused on how the material facilitated their learning by offering visual information about the tests to enhance recall and experiential practice. The ABC is typically administered using a single sheet of paper on which all the items are printed. The VSTEP illustrates the ABC by having a representative image for each item. This feature may support students’ recall of the test. Students also commented on wanting to have an opportunity to practice: “I think anything is better than paper...More active learning than everything on paper and then doing it with a partner” (see comments by Student 5 on the 4SQT; Textbox 2). The role of deliberate practice—one that is purposeful and meaningful—has also been linked with transitioning from novice to expert clinical reasoning [35].

The technology design was consistent with clinical reasoning. Both groups reported that the graphical user interface and flow of the testing sequence aligned with clinical reasoning for the tests. This is in part attributable to the early VSTEP design by clinicians to have the software be consistent with clinical practice. This finding aligns with the TAM perceived usefulness construct. It also highlights the value of including clinicians in practice when designing a teaching tool as they reflect the real-world context of test administration [33]. Graphical representation of the test findings is a result of the digital technology’s capability to measure kinematics using the Kinect camera; the software representation of the information was found to be useful for both the clinician and the patient. Graphical representation of the movement also supports the students’ development of clinical reasoning of movement [36,37] (Textboxes 1 and 2).

Consistent with the taxonomy by Bloom [18] and research on expert and novice clinical reasoning, students required support with both knowledge and application of the tests. The VSTEP supports both basic (test administration) and advanced (test interpretation and application) skills [18]. Novice reasoning was demonstrated with clarifications requested on details of test administration. Expert reasoning by the students was reflected in patient-centered statements [38] (see comments by Student 6 on the 5XSTS; Textbox 2).

Students had lower ratings than faculty on usability. This finding may be due to the faculty members’ tendency to overlook elements of the technology that contributed to negative usability and focus instead on the potential clinical value of the technology. Liu et al [22], using the UTAUT at a large rehabilitation hospital in Canada, reported that clinical usefulness was an important factor to consider when using technology in their clinical practice. However, usability did not significantly contribute to the therapists’ intentions to use technology in their practice [22]. In the VSTEP study, as faculty members had more clinical experience than students, it is possible that they were less likely to dwell on usability concerns, focusing more on the usefulness of the technology.

### Strengths and Limitations

The primary strength and innovation of this study is applying both a quantitative and qualitative methodology. Typically, user studies assess usability by administering an inventory (eg, the System Usability Scale) [39]. The qualitative methodology yielded results that allow for comparison between faculty and students with the rich description of the usefulness theme. In addition, the details of the usability theme are greater than what would be captured using a usability questionnaire. Importantly, this study followed 3 groups of users—physical therapy clinicians who informed the original design and then physical therapy faculty and students. The design of tests that will be used by people in practice may increase their ecological validity. Finally, it is worth noting that the conceptualization and execution of the technology were informed by clinician scientists (JED and JLP), computer artists (AW), human-computer interaction experts (DYW), and biomedical engineers (JLP and HD). The design of the technology with a strong interdisciplinary team and multiple user groups may increase adoption.

There are a couple of potential limitations to this study. First, the focus group facilitation, talk-aloud data collection, and coding of the data were performed by an investigator (JED) who was one of the developers of the technology. Second, both faculty and students knew the investigator. Three steps were taken to control for the potential bias: (1) research activities were conducted in collaboration with other investigators who did not develop the technology (specifically, focus group facilitation scripts were generated by an independent investigator not known to any of the study participants [DW]), (2) data coding was performed by 3 investigators (2 of whom did not collect the data or develop the technology), and (3) a codebook was developed and adhered to by 3 investigators.

### Future Directions

Data from this study will inform the iteration of this technology. Specific features to be added include a rollover description of the test, addition of animations for specific tests that were difficult to understand such as the 4SQT, and adding a trigger to start the 10MWT when the tester is far from the computer.

### Conclusions

A digital education tool was created following a user-centered design; in this case, the users were the clinicians who would administer the tests in practice. Faculty and students then assessed the usefulness and usability of the tool for teaching and learning. This research was performed using mixed methods. The qualitative approach afforded a more detailed understanding of the user than a traditional user study. The process yielded a digital health tool that was deemed useful by both faculty and students. It may be used as a teaching tool as it was consistent with clinical reasoning, supported pedagogy, and ensured reliability of testing. Usability was found to be acceptable for faculty and students, but they raised some concerns. As with any system, there were suggestions to enhance its capabilities and improve existing features.

## Appendix

Multimedia Appendix 1 Script for data collection.

Multimedia Appendix 2 Frequency of Usefulness codes for (A) students and (B) faculty. Positive usefulness codes are represented in the dark color and negative codes are shown in the lighter color. The percentages of positive and negative valence comments are shown in the bars. Frequency calculated based on the number of comments obtained from the inductive coding. 10MWT: 10-Meter Walk Test; 4SQT: 4-Square Test; 5XSTS: 5-Time Sit-to-Stand; 6MWT: 6-Minute Walk Test; ABC: Activities-Specific Balance Confidence Scale.

## Abbreviations

10MWT: 10-Meter Walk Test
4SQT: 4-Square Test
5XSTS: 5-Time Sit-to-Stand
6MWT: 6-Minute Walk Test
ABC: Activities-Specific Balance Confidence Scale
TAM: technology acceptance model
UTAUT: Unified Theory of Acceptance and Use of Technology

## Data Availability

The data sets generated and analyzed during this study are available from the corresponding author upon reasonable request.

## References

[1] Jette DU, Halbert J, Iverson C, Miceli E, Shah P (2009). Use of standardized outcome measures in physical therapist practice: perceptions and applications. Phys Ther.

[2] Salbach NM, Guilcher SJ, Jaglal SB (2011). Physical therapists' perceptions and use of standardized assessments of walking ability post-stroke. J Rehabil Med.

[3] Wedge FM, Braswell-Christy J, Brown CJ, Foley KT, Graham C, Shaw S (2012). Factors influencing the use of outcome measures in physical therapy practice. Physiother Theory Pract.

[4] Rosenberg H, Grad HA, Matear DW (2003). The effectiveness of computer-aided, self-instructional programs in dental education: a systematic review of the literature. J Dental Educ.

[5] Boucher B, Hunter D, Henry J (1999). The effectiveness of computer-assisted instruction in teaching biomechanics of the temporomandibular joint. J Physical Ther Educ.

[6] Plack MM (2000). Computer-assisted instruction versus traditional instruction in teaching human gross anatomy. J Physical Ther Educ.

[7] Ford GS, Mazzone MA, Taylor K (2005). Effect of computer-assisted instruction versus traditional modes of instruction on student learning of musculoskeletal special tests. J Physical Ther Educ.

[8] Sanford MK, Hazelwood SE, Bridges AJ, Cutts JH, Mitchell JA, Reid JC, Sharp G (1996). Effectiveness of computer-assisted interactive videodisc instruction in teaching rheumatology to physical and occupational therapy students. J Allied Health.

[9] Kinney P, Keskula DR, Perry JF (1997). The effect of a computer assisted instructional program on physical therapy students. J Allied Health.

[10] Smith RA, Jones J, Cavanaugh C, Venn J, Wilson W (2006). Effect of interactive multimedia on basic clinical psychomotor skill performance by physical therapist students. J Physical Ther Educ.

[11] Hoglund LT (2015). Mobile devices and software applications to promote learning in a musculoskeletal physical therapy class: a case report. J Physical Ther Educ.

[12] Wilson AB, Brown KM, Misch J, Miller CH, Klein BA, Taylor MA, Goodwin M, Boyle EK, Hoppe C, Lazarus MD (2019). Breaking with tradition: a scoping meta-analysis analyzing the effects of student-centered learning and computer-aided instruction on student performance in anatomy. Anat Sci Educ.

[13] Veneri D (2011). The role and effectiveness of computer-assisted learning in physical therapy education: a systematic review. Physiother Theory Pract.

[14] Gaba DM (2004). The future vision of simulation in health care. Qual Safety Health Care.

[15] Macauley K, Brudvig TJ, Kadakia M, Bonneville M (2017). Systematic review of assessments that evaluate clinical decision making, clinical reasoning, and critical thinking changes after simulation participation. J Physical Ther Educ.

[16] Huhn K, Deutsch JE (2011). Development and assessment of a web-based patient simulation program. J Physical Ther Educ.

[17] Holdsworth C, Skinner EH, Delany CM (2016). Using simulation pedagogy to teach clinical education skills: a randomized trial. Physiother Theory Pract.

[18] Bloom B, Krathwohl D (1956). Taxonomy of Educational Objectives The Classification of Educational Goals.

[19] Davis FD (1989). Perceived usefulness, perceived ease of use, and user acceptance of information technology. MIS Q.

[20] Holden RJ, Karsh B-T (2010). The technology acceptance model: its past and its future in health care. J Biomed Inform.

[21] Venkatesh V, Morris MG, Davis GB, Davis FD (2003). User acceptance of information technology: toward a unified view. MIS Q.

[22] Liu L, Miguel Cruz AM, Rios Rincon AR, Buttar V, Ranson Q, Goertzen D (2015). What factors determine therapists' acceptance of new technologies for rehabilitation – a study using the Unified Theory of Acceptance and Use of Technology (UTAUT). Disabil Rehabil.

[23] Gosine RR, Damodaran H, Deutsch J (2015). Formative evaluation and preliminary validation of kinect open source stepping game. Proceedings of the 2015 International Conference on Virtual Rehabilitation.

[24] Wallach DP, Scholz SC (2012). User-centered design: why and how to put users first in software development. Software for People.

[25] StrokEDGE II documents. Academy of Neurologic Physical Therapy.

[26] Moore JL, Potter K, Blankshain K, Kaplan SL, OʼDwyer LC, Sullivan JE (2018). A core set of outcome measures for adults with neurologic conditions undergoing rehabilitation: a clinical practice guideline. J Neurol Phys Ther.

[27] Hsieh H-F, Shannon SE (2005). Three approaches to qualitative content analysis. Qual Health Res.

[28] Merriam SB (2009). Qualitative Research A Guide to Design and Implementation.

[29] Jensen GM, Gwyer J, Shepard KF, Hack LM (2000). Expert practice in physical therapy. Phys Ther.

[30] Edwards I, Jones M, Carr J, Braunack-Mayer A, Jensen GM (2004). Clinical reasoning strategies in physical therapy. Phys Ther.

[31] Christensen N, Black L, Furze J, Huhn K, Vendrely A, Wainwright S (2017). Clinical reasoning: survey of teaching methods, integration, and assessment in entry-level physical therapist academic education. Phys Ther.

[32] Wainwright S (2019). Andragogy: health professions clinical reasoning transitioning from novice to expert. Clinical Reasoning and Decision Making in Physical Therapy : Facilitation, Assessment and Implementation.

[33] Christensen N, Jensen GM (2019). Expertise in clinical reasoning: uncovering the role of context. Clinical Reasoning and Decision Making in Physical Therapy: Facilitation, Assessment, and Implementation.

[34] Core measure: five times sit-to-stand (5TSTS). Academy of Neurologic Physical Therapy.

[35] Kulasegaram KM, Grierson LE, Norman GR (2013). The roles of deliberate practice and innate ability in developing expertise: evidence and implications. Med Educ.

[36] Skjaerven LH, Kristoffersen K, Gard G (2010). How can movement quality be promoted in clinical practice? A phenomenological study of physical therapist experts. Phys Ther.

[37] Covington K, Barcinas SJ (2017). Situational analysis of physical therapist clinical instructors' facilitation of students' emerging embodiment of movement in practice. Phys Ther.

[38] Resnik L, Jensen GM (2003). Using clinical outcomes to explore the theory of expert practice in physical therapy. Phys Ther.

[39] Brooke J (2013). SUS: a retrospective. J Usability Stud.

