# Supplementary material for: Student and Faculty Perspectives on the Usefulness and Usability of a Digital Health Educational Tool to Teach Standardized Assessment of Persons After Stroke: Mixed Methods Study
Source: JMIR Med Educ. 2023 Aug 10;9:e44361. doi: 10.2196/44361 (PMC10450535; doi:10.2196/44361)
Supplement: Multimedia Appendix 1 [file mededu_v9i1e44361_app1.docx]

Summary of Guiding Questions for Talk-Aloud Procedure

Explain purpose of study: To learn how this technology that was developed for standardized assessment of mobility, balance and coordination of persons post-stroke is easy to understand and use. Indicate that they will be audio recorded. So they should feel free to say anything that comes to mind as they are interacting with the system

Insure that participant knows that we are interested in both what they like and especially what they do not like or is not clear.

Explain the basic layout of the system and the types of tests it can do.

Show how to check that the Kinect camera is on and that it is detecting one or more people.

Explain that every test is organized in a similar way asking some preliminary questions, showing the test set and then the instructions.

Explain (if participant is with a partner) that they will be taking turns being the “clinician” and the “patient”. “We want to make sure that you have a chance to experience the system both ways”

Start with the ABC - Ask them to go through all the screens and complete the test.

Next do the sit-stand (provide the chair)

Follow with the Four Square Test (provide the canes)

Next the 10 MWT

Complete with the 6MWT.

With each test, after they attempt answer questions as needed, as for suggestions to improve, clarify if they say something that is unclear or for them to elaborate

Extra questions for students:

Do you remember in what class you did this test?

Did you have any practice with these tests in your clinicals or the School pro-bono clinic

What do you remember about how these tests were taught in the program?

Extra questions for faculty

What do you teach?

Do you teach any of these tests?

What are your challenges when you teach this content?
