# Supplementary figures and images for: Student and Faculty Perspectives on the Usefulness and Usability of a Digital Health Educational Tool to Teach Standardized Assessment of Persons After Stroke: Mixed Methods Study
Source: JMIR Med Educ. 2023 Aug 10;9:e44361. doi: 10.2196/44361 (PMC10450535; doi:10.2196/44361)

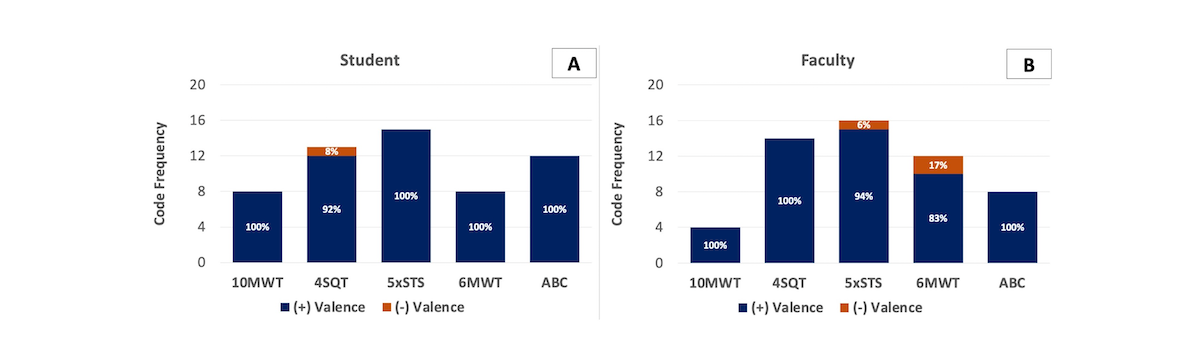

Supplement: Multimedia Appendix 2 [file mededu_v9i1e44361_app2.png]
